# Risk Assessment and Management for Medically Complex Potential Living Kidney Donors: A Few Deontological Criteria and Ethical Values

**DOI:** 10.1155/2011/307130

**Published:** 2011-12-07

**Authors:** Carlo Petrini

**Affiliations:** Bioethics Unit, Istituto Superiore di Sanità, Via Giano della Bella 34, 00162 Roma, Italy

## Abstract

A sound evaluation of every bioethical problem should be predicated on a careful analysis of at least two basic elements: (i) reliable scientific information and (ii) the ethical principles and values at stake. A thorough evaluation of both elements also calls for a careful examination of statements by authoritative institutions. Unfortunately, in the case of medically complex living donors neither element gives clear-cut answers to the ethical problems raised. Likewise, institutionary documents frequently offer only general criteria, which are not very helpful when making practical choices. This paper first introduces a brief overview of scientific information, ethical values, and institutionary documents; the notions of “acceptable risk” and “minimal risk” are then briefly examined, with reference to the problem of medically complex living donors. The so-called precautionary principle and the value of solidarity are then discussed as offering a possible approach to the ethical problem of medically complex living donors.

## 1. Introduction

The debate concerning the most valid approach in bioethics is always open [1]. Whatever ethical perspective one chooses to adopt as a starting point, the analysis must necessarily be founded on two main basic elements: sound scientific facts and the ethical values and principles at stake.

The first part of this article gives a brief overview of the main scientific and clinical information, with no claim to completeness.

Beneficence, nonmaleficence, and autonomy are then briefly discussed, as they are considered the most relevant ethical values involved in organ donation from living persons.

A reliable analysis of these issues should also take account of the relevant statements made by authoritative national, international, and supranational institutions.

Unfortunately, neither scientific data, nor ethical values, nor institutionary documents give precise answers to the ethical questions raised by the problem of explants from medically complex living donors.

Surgical risks should also be weighed against the so-called “minimal risk” and the notion of risk acceptability. Both these issues are briefly discussed, bearing in mind another crucial issue: informed consent.

All of these issues require adequate national policies in order to promote harmonisation of the practices adopted in different transplant centres.

Finally, precaution and solidarity are discussed as being important values in the evaluation of potential living donors: the precautionary principle is not wholly relevant while solidarity is, in every case, one of the most lofty of human attributes.

## 2. Risks for Medically Complex Living Donors: An Overview

Scientific facts are the first basic element for a sound ethical evaluation.

Some “medically complex conditions” do not preclude the possibility of donating a kidney: physical conditions (e.g., hypertension, advanced age, obesity, nephrolithiasis, proteinuria, haematuria, smoking, and drug abuse), familial genetic diseases (e.g., polycystic kidney disease, Alport syndrome, sickle cell trait, and thin basement membrane disease), and physical anomalies in the kidney (e.g., cysts and small renal cell cancers, arterial anomalies, and fibromuscular dysplasia) [2].

An exhaustive review of the medical literature on all the factors that impinge on the health of living kidney donors is beyond the scope of this article. However, a general overview of the literature shows that the scientific data are not clear: “The transplant professional who is faced with counselling a complex donor is confronted with sparse and unsatisfying data about the magnitude of long-term risks” [3].

Some of the difficulties encountered when assessing risks arise not only when medically complex living donors are involved, but also in more general cases of living kidney donations.

An extensive, long-term, and comprehensive examination of donor outcomes performed by Ibrahim and coauthors showed that “the overall evidence suggests that living kidney donors have survival similar to that of nondonors and that their risk of end-stage renal disease (ESRD) is not increased” and that “survival and the risk of ESRD in carefully screened kidney donors appear to be similar to those in the general population. Most donors who were studied had a preserved GFR (glomerular filtration rate), normal albumin excretion, and an excellent quality of life” [4, 5].

A survey conducted at Johns Hopkins University that examined all 80,347 living kidney donations performed in the United States between 1994 and 2009 likewise showed that “living donation is a safe process for donors” [6].

However, some authoritative experts cite “growing concerns about safety of donors” and “the long-term outcome for living donors remains uncertain” [7]: “although the short-term benefit-risk ratio in LDKT (living donor kidney transplantation) is favourable for recipients and donors, the long-term benefit-risk ratio for donors might not be so favourable” [8].

In spite of the presence of some risks, living kidney donation from healthy people is generally considered a safe procedure for donors [9].

The three conditions most frequently encountered in medically complex living donors are hypertension, obesity, and nephrolithiasis. For these conditions too the available data are not univocal.

As regards hypertensive living donors, the largest study was published by Textor and colleagues. The results are intriguing, as they suggest that living kidney donors who adopt a healthy lifestyle may actually benefit in terms of long-term renal function and cardiovascular risk [10, 11]. Some transplant professionals have been reluctant to accept these results as evidence of the long-term safety of accepting hypertensive patients as donors [12] and hypertension continues to be considered, at least in specific conditions, as a contraindication to living donation by the United Network for Organ Sharing (UNOS) and the US Organ Procurement and Transplantation Network (OPTN) [13].

As regards obesity, “increased body mass index has been associated with risk of proteinuria and focal segmental glomerulosclerosis, and kidney function for obese donors also can be harmed indirectly through increased rates of diabetes, hypertension, and the metabolic syndrome” [14].

As regards nephrolithiasis “there are fewer data” [3].

“The shortcomings of the published evidence of long-term risks to living kidney donors with hypertension, nephrolithiasis, and obesity are evident; the data for donors with other risk factors likewise are sparse. Changing demographics, increasing life expectancy, and better therapies for CKD (chronic kidney disease) make quantifying the long-term risk of living kidney donation for complex donors even more problematic” [3].

This situation complicates both risk assessment and ethical opinions.

Regardless of good success rates, recovery rates, and postoperative functionality with one less organ, organ removal causes definite though not incapacitating harm (cutting open the body and removing a healthy organ), and leaves the donor at risk of other complications associated with surgical interventions (Glannon).

## 3. Values at Stake and Conflict between Values

The second element of ethical evaluation is the whole of human values.

The three major ethical principles that should guide transplant professionals in their approach to the issue of complex donors are: beneficence to the recipient, nonmaleficence regarding the donor, and the donor's right to autonomy. However, several conflicts between the different principles may arise.

As regards beneficence, the main contradiction consists in the fact that while it implies a strong argument in favour of ensuring the best medical treatment for patients with renal disease who are waiting for a transplant, the requirement of nonmaleficence (the notion, reaffirmed in every code of medical ethics, that medical professionals have a duty to “do no harm”) conflicts with substantial risks for living donors: in other words, the principle of nonmaleficence is threatened by a scenario in which living donors must undergo a surgical procedure that carries a range of substantial risks, including death. Unlike standard surgery, the removal of a viable organ from a living donor for transplantation is not performed for the therapeutic benefit of the patient: on the contrary, it not only places the donor at risk from the surgical procedure and from postoperative complications, but also implies (in the case of nonregenerative organs) all the disadvantages consequent on the loss of an organ.

While these problems apply to living organ donation in general, they become even more intricate in the case of medically complex living donors.

However, notwithstanding these issues, donations by legally competent volunteers who are aware of the issues at stake are recognised as generous and unselfish acts, making it permissible to subject living donors to risks that may not otherwise be imposed on them. The problem is thus not the existence of risks but the level of acceptability of those risks (discussed below).

“Having beneficence and nonmaleficence in direct opposition is an unusual ethical scenario. One may conceptualise the ‘opposing' demands placed by beneficence and nonmaleficence as weights balancing like a seesaw on a fulcrum of autonomy. In practice, donor autonomy can be respected only by strict adherence to informed consent. Without ensuring valid informed consent on the part of prospective donors, the ethical tension between the responsibility to help transplant candidates and the well-founded concern about harming donors cannot be resolved by appealing to donor autonomy” [3].

At this point the issue of autonomy has to be addressed. An autonomous decision is commonly understood in the medical literature as an act of self determination performed by a competent person. Autonomy (literally: “self-rule”) certainly includes the right not to donate. The question as to whether or not it also includes the right to donate is more complex. In other words, the problem is to decide how far should the respect for autonomy go.

The autonomy of a donor who wants to donate may conflict with the doctor's duty not to harm. However, this does not necessarily happen. As T. M. Wilkinson observes, “It is sometimes thought that living donor transplantation involves a clash between the duty not to harm and the duty to respect autonomy. This is somewhat misleading, however, because of plausible developments of the underlying principles, doing no harm need not speak against live donation and respecting autonomy need not speak in its favour” [15]. In other words, the middle position need not be a compromise between avoiding harm and autonomy: the rule not to do harm does not automatically rule out living donor transplantation, nor does autonomy clearly justify it.

A thorough analysis of the two elements (scientific information and values) also requires a careful reading of documents from authoritative institutions.

## 4. Institutionary Recommendations, Opinions, and Codes

The general recommendations provided in institutionary documents are considered authoritative points of reference for those who have to deal with the problems raised by medically complex living donors. Unfortunately, these statements are often general in their approach and do not provide practical guidance on the specific ethical problems associated with these cases.

This section mentions a few examples from the many available sources. The list (in alphabetical order by country) makes no claims to completeness.

### 4.1. National Bioethics Committees and Commissions

From an ethical perspective, the documents published by National Bioethics Committees are particularly significant.

In [16], the National Health and Medical Research Council of the Australian Government states: “There should not be more than a low probability of serious harm to the donor”, but fails to define either “low probability” or “serious harm.”

As regards the risk-benefit balance, in [17], the Science and Technology Ethics Committee of Quebec, Canada (Commission de l'Éthique de la Science et de la Technologie du Québec, Canada) attaches particular importance to informed consent (“Donor autonomy can be protected by means such as providing donors with all relevant information on the potential risks and benefits and allowing them to withdraw consent confidentially at the time of evaluation”) and to the psychological benefits to the donor (“The benefit to the donor is psychological: saving the life of kin, a child, a spouse, or a friend or significantly improving the quality of life of a loved one is a considerable—and even vital—benefit to the donor.”)

The Danish Council on Ethics does not address the issue of organ donation by living donors in its 2008 report “*Organ donation. Ethical deliberations and recommendations*” [18].

Similarly, in its opinion no. 115 “*Questions d'éthique relatives au prélèvement et au don d'organes à des fins de transplantation*”, the French Comité Consultatif National d'Éthique pour les Sciences de la Vie et de la Santé (National Consultative Ethics Committee for Health and Life Sciences) does not mention the problem of risks for donors [19].

In the information leaflet titled “*Organ donation: the gift of life?*” published by the Irish Council on Bioethics the paragraph “What are the ethical issues associated with live organ donation?” mentions the absence of benefit for living donors, but gives no details of the risks involved [20].

According to the Italian Comitato Nazionale per la Bioetica (National Bioethics Committee) “The removal from living donors should not take place in case of excessive risks for the donor” [21].

The document “*Don d'organes solides par des personnes vivantes. Directives médico-éthiques et recommandations*” by the Académie Suisse de Sciences Médicales (Swiss Academy of Medical Sciences) [22] devotes a paragraph to “donors in particular situations” (“donneurs en situations particulières”) but does not consider particular clinical situations. The “particular situations” are “donneurs souffrants d'un trouble mental, donneurs dont le partennaire de vie refuse le don, donneurs issus d'une autre culture, donneurs qui réfusent la transfusion sanguigne” (donors suffering from mental problems, donors whose partners refuse the donation, donors from different cultural backgrounds, donors who refuse blood transfusions). According to the Swiss Academy “les risques médicaux pour le donneur et pour le receveur doivent être évalués individuellement et présenter un rapport équilibré avec le bénéfice potentiel du don pour les deux parties” (the medical risks for donor and recipient should be evaluated individually and should be equal to the potential benefit of the donation for both parties).

### 4.2. Other Institutions

The “Guiding principle no. 3” of the World Health Organisation's “*Guiding principles on human organ transplantation*” states: “Live donations are acceptable when the donor's informed and voluntary consent is obtained, when professional care of donors is ensured and followup is well organised, and when selection criteria for donors are scrupulously applied and monitored. Live donors should be informed of the probable risks, benefits, and consequences of donation in a complete and understandable fashion; they should be legally competent and capable of weighing the information; they should be acting willingly, free of any undue influence or coercion” [23].

On the subject of living donors, the “*World Medical Association Statement on human organ donation and transplantation*” underlines that donors should “be provided with meaningful and relevant information. Normally, this will include information about (…) the benefits and risks of transplantation” [24], but does not mention the criteria to be used when assessing benefits and risks.

The “*Convention on human rights and biomedicine*” [25] drawn up by the Council of Europe prescribes that “Removal of organs or tissue from a living person for transplantation purposes may be carried out solely for the therapeutic benefit of the recipient and where there is no suitable organ or tissue available from a deceased person and no other alternative therapeutic method of comparable effectiveness” (Article 19). As regards the risks for living donors, the “*Additional protocol to the convention on human rights and biomedicine concerning transplantation of organs and tissues of human origin*” [26] states that: “Before organ or tissue removal, appropriate medical investigations and interventions shall be carried out to evaluate and reduce physical and psychological risks to the health of the donor. The removal may not be carried out if there is a serious risk to the life or health of the donor” (Article 11).

More specific and practical criteria are laid down by the Ethics Committee of the Transplantation Society in “*The consensus statement of the amsterdam forum on the care of the live kidney donor*”. The committee recommends that: “Prior to a live kidney donation to a potential recipient (known by the potential donor or not known in the circumstance of anonymous donation), the donor must receive a complete medical and psychosocial evaluation to include quantification (as available) and assessment of the risk of donor nephrectomy on the individual's overall health” [27]. The committee does not suggest any ethical criteria for assessing the risk, though it underlines the potential complications involved. However, detailed clinical criteria are given in the accompanying report [28, 29].

## 5. Textbooks and Handbooks on Bioethics

Many textbooks and manuals on bioethics contain at least one chapter on the ethics of organ transplantation. However, most either fail to address the problem of medically complex living donors or mention them only briefly.

In the British Medical Association's (BMA) handbook “*Medical ethics today*” [30] a paragraph about organ donation from living doors begins with the question “What level of risk is acceptable?” According to the BMA “it has long been accepted that competent adults are entitled to put themselves at risk to help other people (…). Having accepted that, as a general principle, people may expose themselves to risk for the benefit of another person, are there limits to the extent of that risk? If a surgeon removed, for donation, an individual's hearth, resulting in the patient's inevitable death, any consent would be invalid since the surgeon would commit murder. Apart from such extremes, however, there is no legal restriction on the extent of risk to which individuals may expose themselves in the process of donating organs. Arguably, it is for the competent individual, who has sufficient information and is acting voluntarily, to establish what level of risk he or she is willing to take. Of course, there are also some external controls over such matters. There are independent checks for those who are not genetically related. In addition, surgeons cannot be forced to act contrary to their clinical judgment and any surgeon asked to undertake the operation would need to be satisfied not only that the potential donor was truly competent, informed, and acting voluntarily, but also that the overall benefits of carrying out the procedure outweighed the harms. The BMA believes it is right that the level of harm to which people can give consent is limited but, as in other areas, resists the imposition of inflexible rules. Each case needs to be considered individually and, if the health professionals concerned believe that the risks are too great, the decision and the reasons for it should be sensitively explained to both the potential donor and the recipient” [30].

According to “*The Cambridge textbook of bioethics*” the medical team is responsible for risk assessment for living donors: “Assessments of medical suitability will depend on which organ is being donated and will be carried out by the team physicians” [31]. Moreover, the authors underline that informed consent is crucial: “With respect to informed consent, a donor must be fully and accurately informed about, and demonstrate an understanding of, the risks and benefits of donation as it affects themselves and the recipient.”

“*The Oxford handbook of bioethics*” does not address the problem of risks in living organ donation: it simply mentions a few considerations on the question of resource allocation in US health policies [32].

Likewise, the “*Handbook of bioethics: taking stock of the field from a philosophical perspective*” deals with organ transplantation in a chapter about resource allocation and does not discuss the problem of risks for living donors [33].

In “*A companion to bioethics*”, most of the paragraph on “procurement from the living” deals with the problem of paid donations. As regards risks, the authors merely underline that, in the framework of the Human Tissue Act “there are legal limits to the extent to which harm to others is allowable, even with consent: a surgeon is not allowed to go along with your wish to donate your heart” [34].

According to the manual “*Healthcare ethics. A theological analysis*”: “The functional integrity of the donor as a human person will not be impaired, even though anatomical integrity may suffer” [35].

Many texts and reports on organ donation and transplantation contain at least one chapter on ethical issues, but they usually devote little space to the ethical problems raised by living donations from medically complex donors. For example, the report entitled “*Organ donation: opportunities for action*” by the Committee on Increased Rates of Organ Donation of the Institute of Medicine [36] does not address this specific ethical problem.

## 6. The Problem of Risk Assessment

An acceptable risk-benefit ratio is a prerequisite for a living organ donation: the careful weighing of this aspect is absolutely necessary for a sound ethical analysis of the problem of medically complex living donors.

From a general perspective, two equally crucial constituents of risk should be examined: the probability (“hazard”) and the magnitude (“risk”) of the potential harm, cost, or burden. Although the expression “risk-benefit ratio” is commonly used, a more precise formulation would be “probable risk-probable benefit ratio.” Medical ethics and the law usually adopt a broader definition of “harm” that includes not only physical harm, but also psychological or social harm. For example, parents suffer greatly if their children die, but the burden would be even greater if they could have saved their children by donating an organ and were unable to do so: although the operation would place the parent at risk of harm, failure to operate could lead to an even greater risk of psychological harm.

If the possible mental harm consequent on not donating outweighs the physical harm possibly associated with donating, then an explant from a living donor does not violate the basic ethical rule to do no harm. However, once it is accepted that the physical risk is not the only relevant issue, the problem is to weigh the risks and to decide who is to do the weighing. Surgeons and other medical members of the transplant team might be authorities on the physical risks to the donor, but are probably not best suited to assess the nonclinical risks and how they compare with the potential benefits. Transplant teams that include psychiatrists, social workers, and similar experts probably have a more complete view of the risks and benefits involved. The problem of deciding who is responsible for the “probable risk-probable benefit” assessment belongs to the ethical argument as to who is the “best judge” that recurs in discussions about paternalism: there is a large body of literature on the subject of the “best judge” (dating from the dawn of bioethics as a new branch of knowledge) [37] and who is entitled to take on the role, both in general and in specific circumstances, but this discussion would lead us too far from our subject.

According to a review of these issues “the fundamental ethical problem with accepting complex living donors is limited medical information about the magnitude of potential risk. If data about donor risk are misrepresented, then donor autonomy is undermined. This ethical problem remains challenging even with current attempts to maximise the independence and the integrity of the informed consent process. If a member of the transplant team senses that the informed consent process is not protecting a complex donor adequately, then the centre should refuse the donor. One might ask whether the centre then is permitting paternalism to supersede a donor's right to autonomy, but transplant professionals, as a team, must feel ethically comfortable with the decision to accept or refuse each donor. The conscience of each member of the transplant team merits protection, even if a decision to reject a kidney donor seems paternalistic. Physicians have the right to impose their own sense of acceptable risk in offering procedures to their patients” [3].

For an ethical analysis of the “probable risk-probable benefit ratio,” two other notions should be borne in mind: the “acceptable risk” and the “minimal risk.” Both “minimal” and “acceptable” risks should be evaluated on a scientific basis, but they are more a social choice than a scientific cut-off.

### 6.1. The Notion of “Acceptable Risk”

Most of the literature on the ethical implications of risk acceptability in living donor transplantations concerns the issue of tolerability by recipients.

“It seems that some potential transplant recipients who are nearing death from their organ failure would rationally be willing to accept an organ from someone with a malignancy that appears not to have spread. We need to ask whether the prohibition on procuring organs from persons with cancer is really based on the interest of the potential recipient or whether it is more to serve the psychological needs of surgeons (…). It is hard to imagine an argument supporting a categorical prohibition on transplant from organ donors with cancer” [38]. Maple and coauthors performed a survey “to investigate risk perception relating to living kidney donation, to compare the risk donors would accept with current practice and identify influential factors,” According to their data “kidney donors will accept a higher risk of death than is currently quoted, especially if risks are presented in terms of chance of survival” [39].

The concept of “acceptable risk” is highly subjective and depends on circumstances: the same risk level may be acceptable in some contexts but unacceptable in others. When addressing the issue of “acceptable risk” for medically complex living donors it is necessary to consider each case carefully on its individual merits.

### 6.2. The Notion of “Minimal Risk”

The Royal College of Physicians [40, 41] has used the term “minimal risk” to describe two situations: (1) a small chance of a reaction that is trivial in itself, such as a headache or a feeling of lethargy and (2) a very remote chance of a serious disability or death. In the second situation, the risk to the volunteer should be no greater than that of being a passenger on a scheduled flight.

The US Code of Federal Regulations defines a risk as “minimal” if the “probability and magnitude of harm or discomfort anticipated in the research are not greater in and of themselves than those ordinarily encountered in daily life or during the performance of routine physical or physiological examinations or tests” [42]. This definition is open to discussion, but the debate concerns mainly clinical trials [43].

Nephrectomy by its nature exposes the living donor to risks undoubtedly higher than any “routine” examination; it is nonetheless considered “acceptable” since the available data confirm its essential safety and the quality of postoperative functions.

## 7. Informed Consent

Informed consent is a cardinal issue in medical ethics. It usually requires a competent patient, comprehension of all the circumstances, the absence of coercion, and an objective presentation of meaningful information about the risks and benefits involved [44]. In the case of complex living donors, the first three elements must be ascertained and documented; the problems raised by the other requirement are more challenging: consent for organ donation requires “balancing conflicting ethical obligations” [45].

Some believe that even competent living donors should not be used because their consent can never be determined to be free [46]. This is going too far: authoritative institutions agree that living organ donation is not only ethically acceptable but is an unselfish gesture with a high moral value [47].

There is also the question of whether transplant teams should be obliged to perform operations whenever there are willing recipients and donors. Even if recipients and donors have a right to consent, they do not have a right to insist on donation. It is preferable that teams be allowed a wide margin of discretion. But having the discretion does not tell them how to exercise it.

The adequacy of informed consent should be examined systematically for every donor, though methods to protect the consent process may vary between centres.

It is common practice for both donor and recipient to be evaluated by different experts. The team that evaluates the potential donor should promote only the donor's interests, and care should be taken to avoid external influences on this relationship.

Donors should also be provided with multiple opportunities to rescind their decision to donate during the course of their workup [48]. Some transplant professionals and ethicists have argued that potential donors who opt out of donation for personal reasons should be allowed to represent their decision as having been taken for an undisclosed “medical” reason, in order to protect the potential donor's relationship with the recipient and others [49].

## 8. Need to Harmonise Practices

The disparity between the kidney donor supply and the kidney transplant waiting list has focused attention on living donation as a useful means to increase the supply of organs for transplant candidates.

Many nations have introduced initiatives to promote the donation of living organs. For example, on January 17, 2009 the National Kidney Foundation adopted a “*Position statement on increasing organ donation and transplantation in the United States*” and promoted the “End The Wait” initiative [50], and on November 17, 2008 the Italian National Transplant Centre adopted the “*Documento informativo sul trapianto di rene da donatore vivente*” [51]. (“Information paper on kidney transplantations from living donors.”) The UK, Human Tissue Authority completed a public consultation programme to ensure that the current procedures to assess living donors are sufficiently robust [52] and its annual public meeting featured a discussion on the topic [53].

Initiatives at the national level are important to promote scientifically and ethically sound policies, as well as to encourage improved harmonisation of the practices adopted by different centres: in many countries considerable variations exist regarding how transplant centres present risk, perform psychosocial evaluations, and decide whether to accept medically complex donors. It is important that potential strategies to protect complex donors, as well as the public's trust in living donor transplantation, include, among other elements, the establishment of minimum guidelines for donor evaluation in all centres.

“The decision as to whether a complex potential donor should be accepted should be placed within a well-informed ethical, and legal framework that acknowledges limits on data and provides for independent sources of opinion for prospective donors” [3]. Reese et al. proposed a “medical, ethical, and legal” strategy for complex living donors and summarised it in the following points: “Maximise independence of donor evaluation; improve informed consent process; educate transplant practitioners about legal environment; protect patients from harm; identify which risk factors are clinically important; maximise likelihood that medical complications are identified and treated” [3].

The inclusion of a “donor advocate” on the staff of a transplant centre may offer an opportunity for the donor's interests to be represented by a nonpartisan professional. Ideally, a donor advocate would have a thorough medical understanding of kidney donation, would not answer to the transplant staff, and would be extraneous to incentives that promote the acceptance of complex donors [54], but issues related to recruiting, training, and compensating such advocates could present substantial barriers; no clear model for such a donor advocacy programme is currently available.

Centres could offer potential complex donors an opportunity to discuss their decision in confidence with previous donors. Some potential donors may wish to discuss their decision with previous donors with similar demographic characteristics, such as race or gender. Another possible strategy would be to test donors' knowledge; as an example, standard tests could be used to assess a donor's comprehension of the risks before proceeding with surgery.

Multicentre, long-term studies of health outcomes for complex donors are essential for a clinically significant understanding of the factors associated with risk. Ensuring that long-term healthcare is provided for complex donors could yield the dual benefit of meeting an obligation to donors who have made an altruistic sacrifice and of facilitating the collection of information.

## 9. In Search of Beacons: Precaution and Solidarity

From a general point of view, the overview in the previous paragraphs shows that it is not easy to suggest practical ethical guidelines for the risk assessment of medically complex living donors. This is a typical situation in which each case must be evaluated individually. It is also important to remember that transplantation is typically not a one-party event, but that at least three parties are involved: the donor, the recipient, and the transplant team.

From an ethical perspective, precaution [55] and solidarity [56] have been suggested as attitudes that can help give a sense of orientation when faced with the problem of living donors.

Precaution is a question of duty: it is a crucial principle to be borne in mind when choices about risks have to be made, but it is only partly relevant to the specific problem and is not fully applicable in the case of medically complex living donors.

The notion of “solidarity” is one of the highest moral attributes of human beings—and not only when organ donations from living persons are the issue of the moment.

### 9.1. Precautionary Principle

It is often suggested that the “precautionary principle” is a good point of departure (both scientifically and ethically) when addressing sanitary and health problems.

The precautionary principle holds that even in the absence of scientific certainty that a particular course of action is potentially harmful, cost-effective, and proportionate measures to prevent the risk of serious damage should be adopted.

The principle was formulated in the 1970s, initially in the context of environmental protection [57]. It was later extended to various areas of public health and health protection [58].

Since the 1980s, the precautionary principle has been referred to in numerous institutional documents, conventions, statements, treaties, and regulations [59]. Its most notable affirmation is contained in paragraph 15 of the “Rio Declaration,” proclaimed in 1992 at the conclusion of the United Nations Conference on Environment and Health, which reads, “In order to protect the environment, the precautionary approach shall be widely applied by states according to their capabilities. Where there are threats of serious or irreversible damage, lack of full scientific certainty shall not be used as a reason for postponing cost-effective measures to prevent environmental degradation” [60].

The precautionary principle is therefore an action principle that commits decision makers to take immediate temporary, flexible measures to deal with potential risks in regard to which available scientific data are insufficient, uncertain, or contradictory.

This definition of the precautionary principle clearly shows that several aspects do not apply to the problem of medically complex living donors. The general context within which the precautionary principle was elaborated—environmental policies at the population level—is very different from that of health problems “at the bedside.” However, it is reasonable to suggest that a similar cautionary approach can also be adopted to deal with clinical problems [61].

Some aspects of the precautionary principle (the uncertainty, the potentially serious and permanent risks) do apply to the problem of complex living donors. However, other important aspects are not (at least directly) applicable to organ donation: in particular, transplantation is not a reversible measure. The fact that precautionary policies are usually implemented at the population (rather than individual) level and in an environmental (rather than clinical) setting does not preclude the adoption of a precautionary approach as a criterion for transplantation. Even if the precautionary principle is not strictly enforceable, a cautionary approach is certainly obligatory where medically complex living donors are involved.

### 9.2. Solidarity

Solidarity is one of the highest moral values [62]. Solidarity, considered as a perception of mutual obligations between the members of a community, is deeply rooted in human experience and thinking.

According to “*The short Routledge encyclopedia of philosophy,*” “solidarity exits among a group of people when they are committed to abiding by the outcome of some process of collective decision-making, or to promoting the wellbeing of other members of the group, perhaps at significant costs to themselves. Many regard solidarity as an important political ideal on the ground that it is related to community and fraternity, and conductive to social cohesion and stability” [63].

An attitude of solidarity has accompanied the evolution of much of human thinking and cultural development. Terence spoke of “humanitas,” Virgil of “pietas,” and Seneca of “simpatia” [64]. In Christianity solidarity, or “caritas,” is held in high regard [65].

In moral philosophy solidarity is seen as being open and generous towards other people, putting their best interest before our own, without expecting anything in return. Solidarity provides a remedy to the overemphasis on individualism in contemporary social ethics. Sociality and solidarity principle (justice) include both justice in distribution (“suum quique tribuere”: to each his/her own), and in commutation (“neminem laedere”: do not harm anyone). Solidarity is perceived as a caring and generous attitude towards other people, as putting others' best interests before one's own, with no expectation of reward [64].

A sense of togetherness offers a solid foundation for practices in healthcare.

In many Western countries, equity and solidarity are core values of the National Health Services: according to these models the state is responsible for safeguarding the principles of equal dignity and equal access to citizenship rights [66]. In the European Union, solidarity is also a milestone in health policies [67].

“Establishing the solidarity model is only a minor step for current allocation programs; however, it is a great step for both patients and medical professionals. Patients will benefit for better access to organs, Medical professionals will benefit, because society would reduce the burden of (nonmedical) value decisions under conditions of rationing” [68].

Solidarity is one of the brightest beacons for living organ donations [69, 70]: “The core ethical norm of the medical profession is the principle “do no harm.” The only way that removing an organ from someone seems morally defensible is if the donor chooses to undergo the harm of surgery solely to help another, and if there is sufficient medical benefit to the recipient” [71].

“The right road to follow, until science is able to discover other new forms and more advanced therapies, must be the formation and the spreading of a culture of solidarity that is open to all and does not exclude anyone” [72].

The retrieval of organs for transplantation from medically complex living donors can only be justified as a free and disinterested expression of solidarity towards one's human fellows. Therefore, any policy aiming at increasing the supply of organs should centre on stimulating humanitarian motivations and creating a diffused sensitivity towards the sufferings of people needing organ transplantation [56].
